# The impact of anti-inflammatory drugs on facial odontogenic cellulitis in children: a cross-sectional study in France

**DOI:** 10.1038/s41405-025-00351-7

**Published:** 2025-07-10

**Authors:** Lucille Poure, Caroline Delfosse, Thomas Trentesaux, Fleur Maury, François Dubos, Romain Nicot, Thomas Marquillier

**Affiliations:** 1 Private Practice, Versailles, France; 2 Université Lille, CHU Lille, UFR3S-Odontologie, Odontologie pédiatrique, Lille, France; 3 CHU Lille, Département d’information médicale, Lille, France; 4 CHU Lille, Urgences pédiatriques & maladies infectieuses, Lille, France; 5 Université Lille, ULR2694: METRICS - Évaluation des Technologies de Santé et des Pratiques Médicales, Lille, France; 6 Université Lille, CHU Lille, Department of Oral and Maxillofacial Surgery, INSERM U1008-Advanced Drug Delivery Systems, Lille, France; 7 Université Sorbonne Paris Nord, Laboratoire Éducations et Promotion de la Santé, LEPS, UR 3412, Bobigny, France

**Keywords:** Oral diseases, Paediatric dentistry

## Abstract

**Objective:**

Dental caries is defined by the WHO as a multifactorial non-communicable disease. If left untreated, it can progress to abscesses and then head and neck odontogenic cellulitis. It requires immediate, appropriate, and interdisciplinary treatment. The aim of this study was to draw up an epidemiological profile of these children treated at the Lille University Hospital in northern France and to study the impact of self-medication of anti-inflammatory drugs.

**Materials and Methods:**

A single-centre retrospective, cross-sectional study was conducted on children with odontogenic cellulitis admitted to the paediatric emergency department of the Lille University Hospital between March 2013 and December 2021.

**Results:**

15.3% of the 636 children included had taken nonsteroidal anti-inflammatory drugs before going to the emergency department. The frequency of pain and trismus was higher in children who had taken nonsteroidal anti-inflammatory drugs than in those who had not. Frequency of hospitalisation was higher in children who had not taken nonsteroidal anti-inflammatory drugs than for those who had (70% vs. 57%, respectively; *p* < 0.05). Inversely, the mean length of stay was longer for children who had taken nonsteroidal anti-inflammatory drugs than in those who had not (1.1 vs. 0.8 days, respectively; *p* < 0.05).

**Conclusion:**

This first French epidemiological study on odontogenic cellulitis in children underlines the need to develop multidisciplinary prevention and patient education.

## Introduction

According to WHO, in 2022, 514 million children had dental caries on their primary teeth [1]. Caries are considered the world’s fourth most common disease after cancer, cardiovascular disease, and AIDS. The management of this disease is a real public health challenge, even in industrialised countries [2]. When dental caries are left untreated, it can evolve into an abscess. Virulent bacteria proliferate beyond the dental apex, where they are deprived of oxygen, leading to cellulitis [3]. There are various reasons why dental caries are not treated, such as refusal of treatment, lack of regular check-ups with the dentist, complications resulting from dental trauma, or periodontal disease. In France, there is universal social coverage for patients in disadvantaged economic groups. However, the cost of dental treatment, which is mainly carried out in private practice, limits access to care for some patients [4]. In the Hauts-de-France region, which has a more socially disadvantaged population and a worse oral health situation than the rest of the national territory according to Marquillier et al., the development of odontogenic facial cellulitis under the age of six is a significant marker of precariousness. In addition, access to paediatric dental care is difficult in this region [5].

Head and neck odontogenic cellulitis is an infection of the fatty cellular tissues of the face and neck. Its severity is correlated with its locoregional extent [6]. Septic and thromboembolic complications can compromise the patient’s vital and/or functional prognosis. While acute circumscribed cellulitis is the most common form, it can progress to necrotising fasciitis, which is the most serious form. It therefore requires immediate and appropriate treatment [7]. This infection, which also affects adults, spreads more rapidly in children, leading to an early deterioration of their general condition. The characteristics of primary teeth make them particularly susceptible to dental caries and associated complications [8].

Currently, in France, there is a lack of epidemiological data on head and neck odontogenic cellulitis in children, despite it representing the most serious dental emergency with significant associated risks [9]. A retrospective comparative multicentre study, conducted by Kün-Darbois et al. in 2021 in 18 French oral and maxillofacial surgery departments, provides estimates of 333 cases of head and neck odontogenic cellulitis in 2018 and 2019 and 187 cases in 2020, but all these cases were among adults [10]. A prospective cohort collected over six months in 2006 at Lille University Hospital included 267 patients of all ages, including paediatric patients. The mean age of the patients was 31.3 (±13) years, and 84.9% of them were less than 45 years old, but no paediatric data had been detailed [11]. Similarly, a retrospective study over 10 years carried out at the Clermont-Ferrand University Hospital showed 653 patients aged on average 37 years (range 8 to 88 years) without detailing the epidemiological data in children [12].

The primary objective of this study was to estimate the number of cases of head and neck odontogenic cellulitis in children aged 0 to 16 who were treated in the paediatric emergency department (PED) of a regional university hospital between March 2013 and December 2021.

The secondary objectives were to describe the epidemiological profile of patients, to study the impact of nonsteroidal anti-inflammatory drugs (NSAIDs) on the length of hospitalisation, and to describe the characteristics and management of cellulitis.

## Methods

### Study design and ethics

A single-centre retrospective cross-sectional study was conducted on children with odontogenic cellulitis, admitted at the PED of the Lille University Hospital, between March 2013 and December 2021.

Patients were systematically informed during their first visit to the hospital, via the medical questionnaire, that their data might be processed anonymously for research purposes. The research was declared to the *Commission nationale de l’informatique et des libertés* (CNIL). All procedures performed in the study were in accordance with the ethical standards of the Helsinki declaration. No institutional review board assessment was required because of the retrospective nature of the study, in accordance with French law. We followed the STROBE Guidelines (Appendix).

### Patient selection

Patients were eligible for inclusion if they were between 0 and 16 years of age and visited the paediatric ED of the Lille University Hospital during the study period for a head and neck odontogenic cellulitis. In France, the age of majority is 18. However, specific regulations apply to minors, and the age of 16 is often used as a threshold for certain legal decisions (such as consent to medical treatment). Exclusion criteria were diagnostic error and duplication. Participants were identified through two pathways: the International Classification of Diseases 10th Revision (ICD-10) codes of the medical information systems program (MISP) and the computerised database of diagnostic and therapeutic procedures performed in the paediatric ED. Data were collected from medical records.

France’s health system requires the attribution of at least one diagnosis according to ICD-10 and one treatment according to CCAM to any performed surgical procedure in any patient. CCAM therapeutic codes considered for this research included “evacuation of a collection from the region of the masticatory muscles by intra-oral approach and by facial approach”; “evacuation of a perimaxillary or perimandibular collection by intra-oral approach”; “evacuation of a collection from the region of the masticatory muscles by intra-oral approach”; “evacuation of diffuse cervicofacial and mediastinal phlegmon by cervicotomy”; “evacuation of pelvilingual collection by intra-oral approach”; “evacuation of deep skin and soft tissue collection by direct approach”; “evacuation of pelvilingual collection by intra-oral approach”; and “evacuation of periodontal abscess”. The ICD-10 codes included cellulitis and abscess of mouth and cellulitis and acute lymphangitis of face and neck.

A database was created by a manual search on professional software combined with a computerized search in online data references and reports. The database from both sources was cleaned to eliminate duplicates and then controlled by opening each file in its entirety on the professional software, verifying the correct diagnosis. The final database obtained was then analysed.

### Data collection and aggregation

We collected different data from the medical records using an analysis grid: date of consultation; sex and date of birth; presence of an immunosuppressed condition; medication taken before the visit at the PED; prior consultation with the dentist (before the PED visit); regular check-ups with a dentist; clinical, biological, and radiological signs associated with the disease; specific position of the odontogenic facial cellulitis; status of the causal tooth; cervical extension; main diagnosis code; and type of treatment.

### Statistics

Analysis started with a description of the population of patients with head and neck odontogenic cellulitis. It was a retrospective observational study, which did not require any hypotheses or power calculations. It was not necessary to determine a sample size for descriptive statistics, as this type of study is designed primarily to describe and summarise the characteristics of a population without trying to establish causal relationships or test hypotheses. The study used a sample that was relatively large enough to perform descriptive statistics. Quantitative variables with a normal distribution were expressed as means and standard deviation [SD], while those with a non-normal distribution were expressed as medians and interquartile ranges [Q1; Q3]. Categorical variables were expressed as numbers (percentages). Normality of distribution was assessed using histograms and the Shapiro–Wilk test.

Then, a subgroup analysis was done based on the intake of a NSAID treatment or not. The Mann–Whitney U test was performed to compare non-normally distributed means. We meet the conditions for the statistical tests performed.

Logistic regression analysis was not appropriate for our study because we did not aim to model relationships between the variables studied or predict outcomes. As this was the first study on odontogenic facial cellulitis in children, our main objective was to describe an active patient population.

## Results

### Number and distribution of cases

**Fig. 1:**
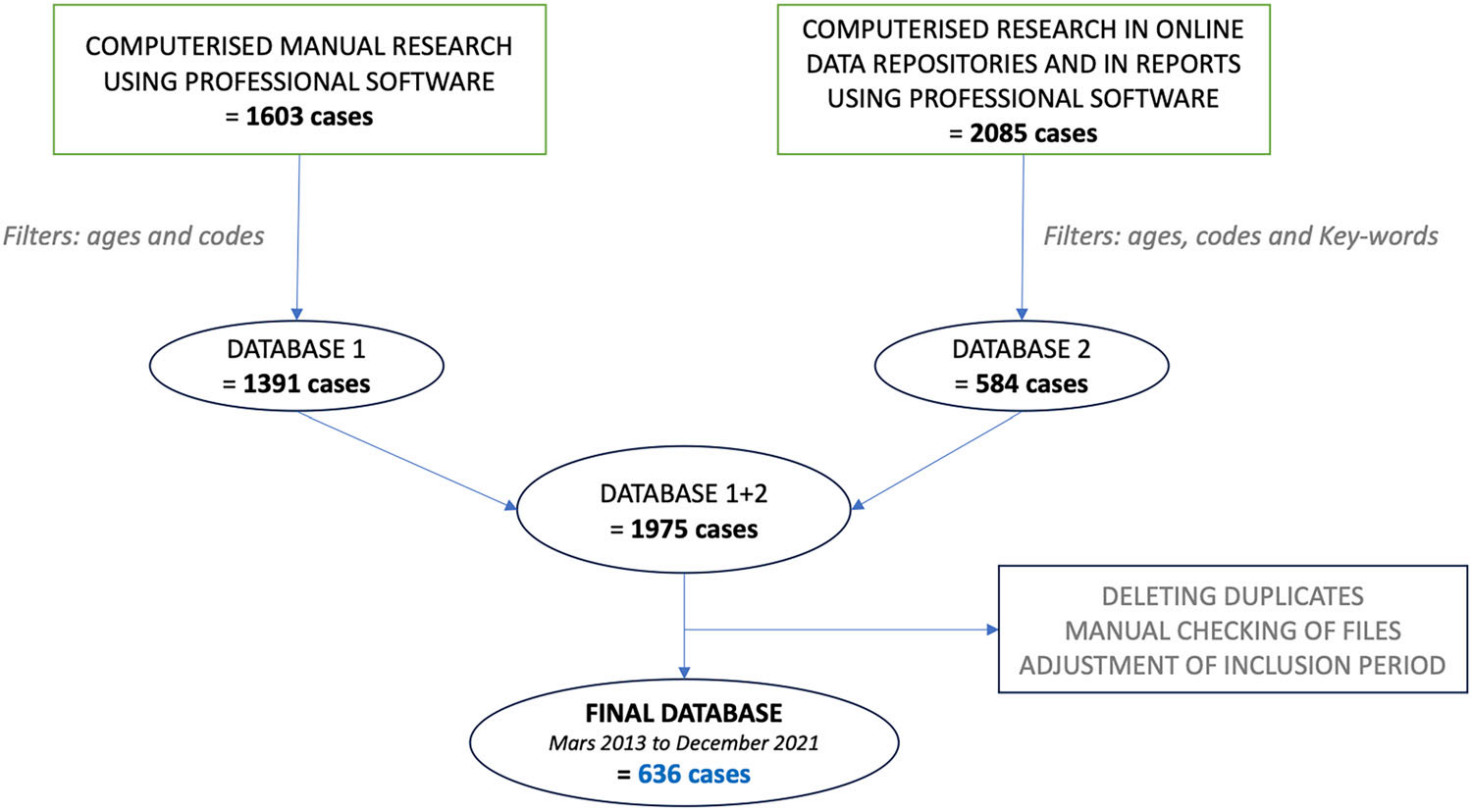
Flow chart of inclusions.

The first available database contained 1603 potential cases. After the filters “age (0–16 years included)” and “diagnostic and billing codes” were applied, 1391 cases were eligible. The second database contained 2,085 potential cases, but 584 cases after applying the “age,” “code,” and “keywords” filters. In all, 1975 cases were therefore eligible. After removing duplicates, manually checking each file, and adjusting the time limits, 636 confirmed cases of head and neck odontogenic cellulitis were included (Fig. 1). There was an average of 71 cases per year (6/month), with 54 recorded in 2013, 83 in 2014, 82 in 2015, 72 in 2016, 113 in 2017, 62 in 2018, 75 in 2019, 57 in 2020, and finally 38 in 2021.

### Epidemiological description

Of the 636 odontogenic cellulitis cases (39% girls [*n* = 245]; male-to-female ratio: 1.6), the 1–5 age group accounted for 29% of the sample (*n* = 185, 69 girls), while the 6–10 age group accounted for 49% (*n *= 309, 116 girls), and the 11–16 age group represented 22% of the sample (*n* = 142, 60 girls). Regarding the seasonality, 163 cases of head and neck odontogenic cellulitis were observed in spring, 156 in summer, 150 in autumn, and 167 in winter. In the sample, there were two patients with an immunocompromised condition. This status was not mentioned in only five cases.

The management of the 636 children before admission to the PED is presented in Table 1. Data on antibiotic and painkiller treatments before admission were available for 46% (*n *= 293) and 53% (*n* = 337) of children, respectively. They are detailed in Table 1. Pain at examination (68%) and facial oedema (73%) were the most frequent clinical signs at presentation (Table 2). Trismus was reported for 20% of children (*n* = 130). A warm and inflamed skin was observed in 7% (*n* = 43) of the children, and 73% (*n* = 463) presented with facial swelling. For 34% (*n* = 156) of the sample, this swelling was associated with hot and inflammatory skin. One child presented with crepitus on the initial clinical examination, and no child presented with depression. The data on signs of exobuccal palpation were missing for 20% (*n* = 129).

**Table 1:** Management before admission to the paediatric emergency department (PED).

Variables	*n* (%)
Decision for the visit to the PED:	
Spontaneous visit (not referred by a dentist)	376 (59)
Referred by a dentist before PED admission	172 (27)
Missing data	88 (14)
Follow-up by a dentist:	
Regular check-up reported	34 (14)
No regular check-up reported	17 (3)
Refusal of care by a dentist reported	18 (3)
Missing data on follow-up by a dentist	567 (89)
Pain management:	
Pain killer level 1	301 (47)
Pain killer level 2	37 (6)
Pain treatment not reported	298 (47)
Antibiotic treatment	293 (46)
Antibiotic treatment not reported	311 (49)
Single antibiotic treatment	270 (92)
Amoxicillin	107
Amoxicillin-clavulanate	148
Macrolide	10
Cephalosporin	2
Synergistin	1
Nitroimidazole	1
Cyclin	1
Bi-antibiotic treatment (Spiramycin + metronidazole or Amoxicillin + metronidazole or Ceftriaxone + metronidazole)	14 (5)
Type of antibiotic not reported	9 (3)

**Table 2:** Clinical description of the children admitted at the paediatric emergency department for odontogenic cellulitis.

Variables	Yes	No	Not reported
Painful examination, *n* (%)	433 (68)	61 (10)	142 (22)
Erythema, *n* (%)	104 (16)	67 (11)	465 (73)
Trismus, *n* (%)	130 (20)	317 (50)	189 (30)
Warm and inflamed skin, *n* (%)	43 (7)	464 (73)	129 (20)
Swelling with hot and inflammatory skin, *n* (%)	156 (34)	351 (46)	129 (20)
Fever, *n* (%)	109 (17)	524 (82)	3 (1)
Dyspnoea, *n* (%)	2 (0)	137 (22)	497 (78)
Dysphagia, *n* (%)	25 (4)	118 (19)	493 (78)
Dysphonia, *n* (%)	4 (1)	55 (9)	577 (91)
Shock (hypotension, tachycardia), *n* (%)	11 (2)	616 (97)	9 (1)
Local warm redness, *n* (%)	199 (31)	308 (49)	129 (20)
Facial oedema, *n* (%)	463 (73)	44 (7)	129 (20)
Crepitations, *n* (%)	1 (0)	506 (80)	129 (20)
Local depression, *n* (%)	0 (0)	497 (80)	129 (20)

At examination, head and neck odontogenic cellulitis were reported equally in each dental sector (Table 3). The details of the dental examination are detailed in Table 3. In our study, 99.8% (*n* = 635) of cellulitis was limited, and one child had diffuse cellulitis.

**Table 3:** Dental examination of the children admitted at the paediatric emergency department for odontogenic cellulitis.

Variables	*n*(%)
Cellulitis in the upper right quadrant	160 (25)
Cellulitis in the lower right quadrant	160 (25)
Cellulitis in the upper left quadrant	153 (24)
Cellulitis in the lower left quadrant	163 (26)
Deciduous tooth*	422 (66)
*Child age: 1–5 years*	*182 (29)*
*Child age: 6–10 years*	*230 (36)*
*Child age: 11–16 years*	*10 (2)*
Permanent tooth*	193 (30)
*Child age: 6–10 years*	*63 (10)*
*Child age: 11–16 years*	*130 (20)*
Serous cellulitis**	307 (48)
Collected cellulitis**	327 (51)

Missing data: *21 and **2.

Imaging, blood tests performed, and medical or surgical management at the ED are presented in Table 4. Overall, 26% (*n *= 168) of children received surgical drainage (local anesthesia and operating room cases), compared to 23% (*n* = 143) who received drainage and dental avulsion (local anesthesia and operating room cases). Of the 143 teeth extracted, 71% were deciduous teeth, while 29% were permanent teeth.

**Table 4:** Management at the paediatric emergency department of the children admitted for odontogenic cellulitis.

Variables	*N* (%)
Imaging (first-line):	377 (59)
Panoramic radiography, or orthopantomogram	329 (87)
Computed tomography	45 (12)
Retro-alveolar radiography	3 (1)
Imaging (second-line):	86 (14)
Computed tomography	81 (94)
Panoramic radiography	4 (5)
Retro-alveolar radiography	1 (1)
Blood test	72 (11)
Dental care:	
Medical treatment	324 (51)
Referred secondary to a dental surgeon for treating the causal tooth	203 (63)
Surgical treatment under local anaesthesia in the ED (short circuit)	134 (21)
Age: 1*–*5 years	24 (18)
Age: 6*–*11 years	65 (49)
Age: 11*–*16 years	45 (34)
Surgical drainage only	130 (97)
Surgical drainage + immediate extraction of the causal tooth	4 (3)
Surgical treatment under general anaesthesia in the operating room	178 (28)
Age: 1*–*5 years	70 (39)
Age: 6*–*11 years	74 (42)
Age: 11*–*16 years	34 (19)
Surgical drainage only	38 (21)
Surgical drainage + immediate extraction of the causal tooth	139 (78)

*ED* Emergency Department.

Among hospitalised patients (32% [*n* = 204]; girls: 39%), 6% (*n* = 13) had received surgical treatment under local anaesthesia (drainage +/- extraction), 87% (*n* = 177) had received surgical treatment under general anaesthesia (drainage +/- extraction), and 7% (*n* = 14) had only received medical treatment. Of the 190 children who received a surgical procedure, under local anaesthesia (*n* = 13) or general anaesthesia (*n* = 177), 25% (*n* = 47) were drained, and 75% (*n* = 142) received drainage and avulsion. For 77% of patients (*n* = 152), the length of hospitalisation was under 72 h (Table 5). Regarding the length of hospitalisation, the stay is mostly over 24–48 h for children aged 1–5 and 6–10 years, while for those aged 11–16 years, it is mostly over 72 h.

**Table 5:** Length of hospitalisation by age and NSAIDs.

Variables	*N* (%)
1–5 years	74 (37)
<24 h	3 (4)
24 – 48 h	40 (54)
48 – 72 h	22 (30)
>72 h	9 (12)
6–10 years	84 (43)
<24 h	2 (2)
24 – 48 h	33 (39)
48 – 72 h	29 (35)
>72 h	20 (24)
11-16 years	40 (20)
<24 h	2 (5)
24 – 48 h	7 (18)
48 – 72 h	14 (35)
>72 h	17 (42)
NSAIDs	97 (15)
Frequency of pain	77 (79)
Frequency of trismus	29 (30)
Frequency of hospitalisation	55 (57)
No NSAIDs	539 (85)
Frequency of Pain	356 (66)
Frequency of trismus	102 (19)
Frequency of hospitalisation	377 (70)

### NSAIDs

Of all included children, 15% (*n* = 97) had taken NSAIDs before going to the PED (Table 5). Both groups were comparable for demographics and clinical data except for pain and trismus. The frequency of pain was higher in children who had taken NSAIDs than in those who had not (79% vs. 66%, respectively; *p* < 0.01). Similarly, the frequency of trismus was higher in children who had taken NSAIDs than in those who had not (30% vs. 19%, respectively; *p *< 0.05). Both groups were comparable for odontogenic cellulitis staging and for modalities of treatment (medical, surgical under local or general anaesthesia). The frequency of hospitalisation was higher in children who had not taken NSAIDs than in those who had (70% vs. 57%, respectively; *p* < 0.05). Inversely, the means length of stay was higher for children who had taken NSAIDs than in those who had not (1.1 vs. 0.8 days, respectively; *p* < 0.05). The frequency of hospitalisations in children treated with NSAIDs was not statistically different between children with and without concomitant antibiotic treatment (50% vs. 32%, respectively; *p* = 0.09). As a reminder, a hospitalisation corresponds to a stay in hospital (counted for administrative formalities) and can be either ambulatory (a day’s stay for a treatment involving medication) or conventional (several days). This is different from a simple consultation.

## Discussion

In addition to acknowledging the typical biases associated with retrospective studies, it is important to point out that the analysed data were extracted from medical records and then clearly recorded. However, it is possible that certain information was forgotten or incorrectly filled in and therefore not recorded and analysed when the file was completed. This is an example of information bias, also known as measurement bias or classification bias. One possible limitation of the study was the sometimes-high proportion of data not filled in for certain items. Missing data in medical records represent a bias in research and should be a point of vigilance. They can lead to over-representation of certain data. In addition, these gaps create a selection bias, where patients without data may have specific characteristics that are not represented, leading to incorrect generalizations. As this is the first French retrospective study in children, this aspect could be better addressed in the next prospective study.

### Number and distribution of cases

There were 636 cases of head and neck odontogenic cellulitis recorded in this study over 8 years. The French study conducted in 2021 in 18 oral surgery and maxillofacial surgery departments reported, notably in Lille, figures of 95 cases in 2018, 90 cases in 2019, and 50 cases in 2020 of head and neck odontogenic cellulitis in adults [10]. Thus, compared to adult data, the mean annual number of odontogenic cellulitis cases managed in our centre is high in children (except for 2020, due to the COVID-19 pandemic).

Other international studies have recorded a higher number of cases of head and neck odontogenic cellulitis in the paediatric population than in adults. One such study, carried out in China in 2003, reported 56 cases in that year, while a study carried out in Malaysia reported 153 cases over 3 years, i.e., 51 cases per year [13, 14]. With an average of 71 cases per year, we therefore have a higher number of cases in Lille than those found in those two studies. However, it is difficult to compare these figures in so far as the healthcare systems (particularly access to prevention, diagnosis, and treatment) are not comparable.

### NSAIDs

Head and neck odontogenic cellulitis is a serious infection that can be life-threatening. Among the risk factors, the use of NSAIDs is frequently reported, and these should therefore be used with caution [15]. In fact, NSAIDs may lead to an increase in infections in children [16]. In our study, the use of NSAIDs was associated with longer hospital stays. However, when combined with antibiotics, it may reduce the frequency of hospitalisation. Previous studies have shown that the severity of facial or head and neck odontogenic cellulitis was not correlated with initial anti-inflammatory treatment [11].

### Epidemiological description

Boys predominated in the study by Kara et al.; with a boy/girl ratio of 1.4:1, as in our study (1.6:1), whereas in the study by Lim et al., girls were slightly more affected than boys, representing 52.9% of cases [13, 14]. The predominance of boys might be explained by the fact that they attach less importance to their oral hygiene than girls. Additionally, a study published in 2021 indicated that men are more likely to neglect their oral health, consult dentists less regularly than women, and have a higher risk of oral pathology [17]. The most represented age group in our study was children aged 6–10, followed by those aged 1–5. Studies confirm that the 6–12 age group is the most affected in cases of dental infection, followed by the 0–5 age group [9, 18, 19]. This could be explained by the fact that children aged between 6 and 10 brush less effectively than older children yet are often no longer supervised by parents, who believe them to be autonomous, resulting in the appearance of carious lesions that can lead to cellulitis if left untreated. In the 0–5 age group, the high prevalence of dental infection may be explained by inadequate oral hygiene combined with the consumption of sugary products, leading to the development of early childhood caries. A study that examined the oral cavities of 336 children aged 2 to 5 years old reported that the prevalence of caries at age five was significantly linked to brushing teeth less than twice a day during the nursery years, as well as difficulties when brushing was carried out (54). In our study, similar results were reported during all four seasons, although there was a very slight increase in cases of head and neck odontogenic cellulitis in the winter. These results suggest that the potential seasonal effects found in certain studies, such as the one conducted in Germany in the 1970s or the one in Dijon, France, in the early 2000s, remain controversial in the literature [20].

Our study corroborates the fact that the clinical signs of cellulitis, in order of importance, are pain, oedema, and, less frequently, trismus and fever [14, 21]. However, our study reports a lower proportion of febrile children than other studies. This could be explained by the fact that more than half of the children in our study were taking analgesics (paracetamol also being an antipyretic) before arrival at the PED [22]. Primary teeth were more often the causal teeth in head and neck odontogenic cellulitis, a finding consistent with existing studies [9, 23]. For most children in the 1–5 age group, the causal teeth were all deciduous teeth. In the 11–16 age group, permanent teeth were the main cause of cellulitis, which seems consistent at this age. In the 6–10 age group, deciduous teeth were more likely responsible for cellulitis. This age-related divergence could be explained not only by the fact that treating young children is more complicated (either because of the child’s lack of desire to cooperate or because the practitioner says they are not competent to treat a young child) than treating older children but also by the still widespread belief that primary teeth do not need to be treated because they will eventually fall out and make way for permanent teeth [24].

Panoramic radiography, as in other studies, was the examination most frequently performed as a first line of imaging [14]. This would appear to be consistent with the fact that few general EDs have facilities for retro-alveolar radiography and that CT scans are performed when there is a suspicion of extension, and verification is then necessary. CT scans can be performed if cellulite has extended. CBCT can also be useful to assess extension and prior to a local dental procedure. This type of imaging is widely used by dentists and is often available in general practices. Few biological blood tests were found in this study, as they are not routinely performed and recommended in clinical practice in the paediatric population.

One-third of the children in our study required hospitalisation, compared with just over half in the study by Lin et al. (53.6%). The duration of hospitalisation in our study was relatively short compared to certain other studies that reported average hospitalisation times of around 5 days and was in agreement with others that found an average length of stay close to ours [9, 13, 19, 21, 22]. The work of some authors suggests that the shorter average length of stay in our study may be explained by the rapid removal of the tooth causing the infection during hospitalisation [19, 25]. Doll et al. showed a statistically significant relationship between duration of hospital stay and different age groups (p < 0.001): the length of hospital stay of patients aged 14 to 17 years was twice that of patients aged under six [25]. Considering that in our study the longest length of stay was 2–3 days, we confirm that three quarters of children aged 11–16 experienced long hospital stays, compared to 58.3% of children aged 6–10 and 41.5% of children aged 1–5. We might assume that time to recover is longer in older than in younger children, or that head and neck odontogenic cellulitis is more significant when the tooth involved is a permanent tooth.

## Conclusion

This study represents the first comprehensive epidemiological analysis of head and neck odontogenic cellulitis in children in France, highlighting the significant active patient file of this condition among the paediatric emergency department. The findings highlight the urgent need for improved oral health prevention strategies and better access to dental care for children, particularly in socially disadvantaged populations.

Additionally, the research reveals that the use of non-steroidal anti-inflammatory drugs (NSAIDs) prior to hospital admission may influence the clinical outcomes, including increased pain and longer hospital stays. These insights are crucial for healthcare professionals to enhance the management of odontogenic infections in children and foster collaboration among dental and medical practitioners to optimize patient care. Our results suggest that further investigation into the use of NSAIDs and other factors is warranted. Logistic regression analyses could be conducted to further investigate the relationships between the variables.

## Supplementary information

STROBE Checklist - Appendix 1

## Data Availability

Raw data are available on OSF repository: 10.17605/OSF.IO/S4572.
